# Supplementary material for: A Qualitative Study: Mothers’ Experiences of Their Child’s Late-Onset Pompe Disease Diagnosis Following Newborn Screening
Source: Int J Neonatal Screen. 2022 Jul 19;8(3):43. doi: 10.3390/ijns8030043 (PMC9326644; doi:10.3390/ijns8030043)
Supplement: Supplementary file 1 [file IJNS-08-00043-s001.zip › IJNS-1711811-supplementary.pdf]

Supplemental information S1

Interview Guide

Hello, my name is Kaylee Crossen. I am a genetic counseling graduate student at the University of Cincinnati and Cincinnati Children's Hospital. I am conducting a study to learn parents' experiences receiving a diagnosis of Late-Onset Pompe Disease (LOPD) on newborn screening and their experiences with their child's medical monitoring. Thank you for your interest in this study.

This interview will be conducted in private and will be audio-recorded. The recording will be transcribed. To protect your privacy, you will be assigned a study ID that will be associated with the information you provide. We will remove your name and your child's name and any other identifiers from the transcripts. Only the research team will have access to the documents linking your study ID number and your name.

Demographic questions and open-ended questions will be asked in this interview. There are no right or wrong answers. You do not have to answer every question. You may withdraw from the study at any point if you do not wish to participate.

The answers you provide will help us understand parents' experiences of having a child with LOPD. While there is no direct benefit to you, the information you provide may help other families of children with LOPD or providers.

In order to participate in this study, you must meet the eligibility criteria. I have a few questions to ask you to help determine this.

1. Are you a parent or legal guardian of a child who was diagnosed with Late-Onset Pompe Disease on newborn screening?
2. Are you at least 18 years of age?
3. Has your child started ERT (Enzyme Replacement Therapy) for their LOPD?
4. Does your child have cardiomegaly and cardiomyopathy?

If they meet criteria, continue. If they do not meet criteria, "Thank you for your interest, but unfortunately we cannot continue with the interview."

The interview will take approximately one hour. Is this a good time to speak? (If no, ask for a better date and time)

Do you consent to participate in the interview?

Do you have any questions for me before we begin?

Okay to start, I am going to ask you for brief demographic information to get to know you better.

Demographics:

1. How old are you?
    a. Enter age
2. What is your sex?
    a. Male
    b. Female
    c. Prefer not to answer
3. What is your race?
    a. Caucasian

    b. African American
    c. Asian
    d. Hispanic
    e. Other
4. What is your marital status?
    a. Married
    b. Divorced
    c. Single
    d. Widowed
    e. Other
5. What is your highest level of education?
    a. Some High School
    b. High School Degree
    c. Some College
    d. College Degree
    e. Some Graduate School
    f. Graduate Degree or More
6. How many people, including yourself, live in your household?
    a. Enter Size
7. Does your child with LOPD have public or private insurance?
    a. Public
    b. Private
    c. Unknown
8. How old is your child who was diagnosed on newborn screening with LOPD?
    a. Enter Age
9. If you have older children than your child with LOPD, how many were screened for LOPD?
    a. Enter number
10. Of your other children screened for LOPD, how many were diagnosed?
    a. Enter number
    b. How old are they?
        i. Enter age
11. Which state do you live in currently?
    a. Enter State:
12. Which state was your child with LOPD born in?
    a. Enter State:
13. How far do you live from the center where your child receives care for LOPD?
    a. Enter distance (in miles):

Interview:

Diagnosis questions:
Now I am going to ask you about your experience with the diagnosis of LOPD for your child. These questions will be open-ended, and you can share as much or as little as you'd like.

Can you first tell me a little bit about your family such as who makes up your family?

Next, I will ask you about your experiences with Late-Onset Pompe Disease.

I understand your child was diagnosed with Late-Onset Pompe Disease. I would like to know more about how you learned about the diagnosis. Can you tell me more about your experience receiving the diagnosis?

- Probes:
  - How did you learn about the diagnosis?
  - What were you feeling?
    - Compared to now

What does this diagnosis mean to you?

What does the diagnosis mean for your child? – (depending on child's age)

Has this experience influenced how you see your child and their health?

Medical Monitoring Plan Questions:

I will now ask you some specific questions about how your child is monitored for LOPD. The following may not all be familiar to you and in no way is this an extensive list of how your child should be followed, as some of these items may not be appropriate for your child and others with LOPD.

1. What providers follow your child for LOPD?
   - ☐ Geneticist
     - i. How often in person? ___________
     - ii. How often by telemedicine? ___________
   - ☐ Cardiologist
     - i. How often in person? ___________
     - ii. How often by telemedicine? ___________
   - ☐ Pulmonologist
     - i. How often in person? ___________
     - ii. How often by telemedicine? ___________
   - ☐ Pediatrician
     - i. How often in person? ___________
     - ii. How often by telemedicine? ___________
   - ☐ Other
     - i. ___________
     - ii. How often in person? ___________
     - iii. How often by telemedicine? ___________

Often when your child sees a provider for LOPD, they perform a physical exam, specifically they want to look at their muscle strength. Typically, they look at sitting, head control, walking, and climbing stairs. The next few questions will ask who performs these examinations.

2. Who conducts physical exams for your child for LOPD?
3. How often does your child receive a physical exam for LOPD?
   - ☐ Once a year
   - ☐ Once every 6 months
   - ☐ Once every 3 months
   - ☐ Other________________
4. How comfortable are you with this provider conducting a physical exam for LOPD?

5. Do you think other providers should be assessing muscle strength?

Now I will ask you about a few more tests your child receives such as blood and urine screening, heart monitoring, and other assessments.

6. Has your child received blood tests?
    - ☐ Yes
        - i. Is your child currently receiving blood tests?
            - ☐ Yes
            - ☐ No
                - a. How many blood tests has your child had in the past?
                    - i. ________________
        - ii. Are they checking for:
            - ☐ CK/CPK levels (Creatine Kinase)
            - ☐ Kidney/Liver function (Renal/Hepatic function)
            - ☐ Other ________________
            - ☐ Unknown
        - iii. How Often?
            - ☐ Once a year
            - ☐ Once every 6 months
            - ☐ Once every 3 months
            - ☐ Other________________
    - ☐ No
    - ☐ Unknown
7. Has your child received urine tests?
    - ☐ Yes
        - i. Is your child currently receiving urine tests?
            - ☐ Yes
            - ☐ No
                - a. How many urine tests has your child had in the past?
                    - i. ________________
        - ii. Are they checking for:
            - ☐ Hex4 (Glucotetrasaccharides)
            - ☐ Other ________________
            - ☐ Unknow
        - iii. How Often?
            - ☐ Once a year
            - ☐ Once every 6 months
            - ☐ Once every 3 months
            - ☐ Other________________
    - ☐ No
    - ☐ Unknown
8. Has your child received ECGs or EKGs?
   An ECG/EKG measures the heartbeat and can identify differences in heart rhythm.
    - ☐ Yes
        - i. Is your child currently receiving ECGs/EKGs?
            - ☐ Yes
            - ☐ No

a. How many ECGs/EKGs did your child have in the past?

i. ________________

ii. How Often?

- ☐ Once a year
- ☐ Once every 6 months
- ☐ Once every 3 months
- ☐ Other_________________

- ☐ No
- ☐ Unknown

9. Has your child received an echo?
An echo is an ultrasound of the heart which helps identify the shape and parts of the heart.
   - ☐ Yes
     i. Is your child currently receiving echos?
        - ☐ Yes
        - ☐ No
          a. How many echos did your child have in the past?
             i. ________________
     ii. How Often?
        - ☐ Once a year
        - ☐ Once every 6 months
        - ☐ Once every 3 months
        - ☐ Other_________________
   - ☐ No
   - ☐ Unknown
10. Has your child received a Chest X-rays?
   - ☐ Yes
     i. Is your child currently receiving Chest X-rays?
        - ☐ Yes
        - ☐ No
          a. How many Chest X-rays has your child had in the past?
             i. ________________
     ii. How Often?
        - ☐ Once a year
        - ☐ Once every 6 months
        - ☐ Once every 3 months
        - ☐ Other_________________
   - ☐ No
   - ☐ Unknown
11. Has your child received a Physical Therapy (PT) assessment?
   - ☐ Yes
     i. Is your child currently receiving PT assessments?
        - ☐ Yes
        - ☐ No
          a. How many PT assessments has your child had in the past?
             i. ________________
     ii. How Often?
        - ☐ Once a year
        - ☐ Once every 6 months

- ☐ Once every 3 months
- ☐ Other________________

- ☐ No
- ☐ Unknown

12. Has your child had a sleep study?
    - ☐ Yes
        i. Is your child currently receiving sleep studies?
            - ☐ Yes
            - ☐ No
                a. How many sleep studies has your child had in the past?
                    i. ________________
        ii. How Often?
            - ☐ Once a year
            - ☐ Once every 6 months
            - ☐ Once every 3 months
            - ☐ Other________________
    - ☐ No
    - ☐ Unknown
13. Has your child received hearing screening?
    - ☐ Yes
        i. Is your child currently receiving hearing tests?
            - ☐ Yes
            - ☐ No
                a. How many hearing tests has your child had in the past?
                    i. ________________
        ii. How Often?
            - ☐ Once a year
            - ☐ Once every 6 months
            - ☐ Once every 3 months
            - ☐ Other________________
    - ☐ No
    - ☐ Unknown
14. Has your child received feeding and swallowing testing?
    - ☐ Yes
        i. Is your child currently receiving feeding and swallowing tests?
            - ☐ Yes
            - ☐ No
                a. How many feeding and swallow tests has your child had in the past?
                    i. ________________
        ii. How Often?
            - ☐ Once a year
            - ☐ Once every 6 months
            - ☐ Once every 3 months
            - ☐ Other________________
    - ☐ No
    - ☐ Unknown
15. What other tests, assessments, or examinations has your child received?

Thank you for that information. I am now going to ask you more questions about the monitoring plan that has been recommended for your child, specifically about your experiences. Can you tell me more about your experiences with monitoring your child's health?

How do you feel about how your child is monitored for LOPD?

What are your thoughts about the benefits of how your child is currently monitored?

- Probe on whether benefits are tangible and/or emotional

What are your thoughts about the drawbacks of how your child is currently monitored?

- Probe on whether drawbacks are tangible and/or emotional

What barriers do you and your family face with monitoring your child?

- Probe on culture and distance and trust

Tell me about the support you have for managing the diagnosis, your child's health, and monitoring plan.

I am going to continue to ask about the monitoring plan but now I would like to get insight on what you think will work best for your family and child.

Are there any changes you would like to see in how your child is followed? If so, what and why?

Do you have any suggestions you would like to pass on to other parents or to the healthcare providers who follow children with LOPD?

Is there anything else you would like to share that you think is important for providers to know that I have not asked you?

Thank you for your time and participation in this interview!

Supplemental information S2

Codebook

Category	Code	Subcode	Description
Diagnosis	Experience	Difficult News	The family reported they received difficult news
		Dx Communication	Ways in which parents were communicated with about diagnosis
		Experience	General experience parent went through
		Sibling Testing	Experience with testing other children
		Waiting	Family discussed the wait it took for diagnosis
	Knowledge	Better Understanding	Parent has better understanding from beginning
		Child Knowledge	Knowledge that the child has about visits/Pompe
		Genetic Terms	Parent discuss specific terms related to genetics
		Google	Parent gained knowledge from Google
		Lack of Understanding	Parent had a lack of understanding about diagnosis process
		Parent Knowledge	Knowledge about Pompe that parent has or lack of knowledge they had/have and what they have researched
		Partner Communication	Parent relays information to partner
		Provider Knowledge	Knowledge providers knew about Pompe
	Labeling	Label	Parent talks about labelling diagnosis in general
		Label: risk factor	Parent sees diagnosis as a risk factor
Hypervigilance		High Alert	in vivo to explain hypervigilance
		Hypervigilance	Parent pays extra attention to child between visits and pays extra attention to their child's health and development
		Protective	Parents are more protective of child
Anxiety/Worry		Anxiety	General anxiety
		Anxiety: future	Parent is anxious about future for their child
		Anxiety: heart	Parent has anxiety for child's heart involvement
		Anxiety: information	Parent feels that too much information is anxiety inducing

		Anxiety: labs	Parents have anxiety about labs
		Anxiety: milestones	Parents worry about if child will meet milestones
		Anxiety: onset	Parents have anxiety about when symptoms will occur
		Anxiety: telling children	Parents are nervous about telling child and siblings
		Anxious Heart	in vivo
		Unnecessary Worry	Parents worry more than they should while child is asymptomatic
		Worry: insurance	Parent fears what criteria insurance will cover ERT
		Worry: more intervention	Parent is worried child needs more monitoring or interventions
		Worry: older children	Parent worries other children may be affected after initial diagnosis on NBS
		Worry: symptom onset	Parent worries about disease onset
Uncertainty		Anticipatory Grief	Parent experience grief with asymptomatic children
		Uncertainty	General uncertainty
		Uncertainty: dx	Parent has uncertainty about what diagnosis would be
		Uncertainty: standard of care	There is uncertainty about the standard of care and what is recommended
Emotions	Negative Emotions	Desperation	Parents feel desperate
		Devastation	Parents are devasted and feel devastation
		Disheartening	in vivo, response to provider knowledge
		Exhausted	Parents were exhausted during diagnosis process
		Frustration	Parents feel frustration
		Grief	Parents grieve with new diagnosis and while waiting
		Heartbroken	Parent feels heartbroken (in vivo)
		Scared	Parents were scared
		Shock	Parent felt shock
		Stressful	Parent was stressed
		Traumatic	Parents report experience of diagnosis to be traumatic
		Confident	Parent feels confident with care and future

	Positive Emotions	Hope: no progression	Parent has hope from child being asymptomatic
		Hope: other PD children	Parent has hope from seeing other PD children doing well
		Hope: research	Parent has hope that research will bring better surveillance and treatments
		Hopeful	Parent has hope
		Peace	Parent was able to gain peace
		Relief	Parent is relieved about Late onset vs Infantile onset
		Thankful	Parent is thankful and grateful
	Emotion Changes	Emotional Improvement	Parent has better emotions since beginning
		Reassurance	Parent has been reassured
Monitoring	Physical Exam Opinions	Geneticist Confidence	Parent is confident with geneticist performing physical exams
		Geneticist Doubt	Parent is not confident with geneticist performing physical exams
		Neuromuscular Confidence	Parent is confident with neuromuscular doctor performing physical exams
		PCP Doubt	Parent is not confident with PCP performing physical exams
		PT Confidence	Parent is confident with PT performing physical exams
	Experiences with Monitoring	Advocacy	Parent advocates for child and their care
		Collaboration	Parent and Provider collaboration for their child's monitoring
		No Monitoring	Parent does not want to monitor while asymptomatic
		Normalization	Parent feels that monitoring is just a part of their normal life
		PCP Educated	PCP educated themselves on Pompe
		Preparedness	Parents feel prepared and know what will happen during visits
		Telehealth	General experience with telehealth
Benefits of Monitoring	Emotional	Comfort	Comfort comes from monitoring child for symptoms
		Safety	Safety comes from monitoring child for symptoms

	Tangible	Benefit: early detection	Parents see the benefit of early detection and starting treatment early
		Benefit: multiple providers	Parents like that there are multiple providers following child
		Benefit: providers	Providers make the appointments easier and provide information well
		Minimization	Parent's providers are eliminating unnecessary tests
Drawbacks of Monitoring	Emotional	Drawback: asymptomatic	There is many visits while asymptomatic
	Tangible	Drawback: older children	Older children have difficulties going to appointments and missing school
		Drawback: provider understanding	Some providers do not understand their need for Pompe
		Drawback: repeated tests	There are many tests that are repeated while normal
		Drawback: schedule	Between multiple kids, appointments and school it is difficult to schedule
		Drawback: time	The time appointments take is a drawback
		Drawback: waiting	Parent does not like waiting for initial visit of other visits
		Drawback: worry	Parents worry about every visit
Barriers and Challenges		Barrier	Parent discusses what barrier they face
		Challenge: insurance	Parent had difficult with testing or monitoring being covered
		Challenge: provider	Parents did not agree or work well with providers
		Challenge: telehealth	Telehealth is challenge for parent to inform specialist
		Challenge: work	Parent takes off work for visits
Desires	Changes for Diagnosis	Recommendation: initial news	Parents would like to see change in how the initial news is given
		Recommendation: join community sooner	Parents would like to get involved with support groups sooner
		Recommendation: mindful	Parents would like providers to be mindful of situation (trainee was not experienced)
	Changes for Monitoring	Change: less visits	Parents would like less visits to decrease how overwhelming they will be if child is still asymptomatic

	Change: provider communication	Parents would like providers to communicate more about child
	Recommendation: anticipatory guidance	Parents would like more information about other older children with LOPD
	Recommendation: Duke	Parent recommends that other parents follow with Duke
	Recommendation: prepare	Parent would like providers to be more prepared to see child
	Recommendation: proactive	Parents would like other parents to be proactive about child's health
	Recommendation: prognosis	Parents would like more prognosis information
Coping Strategies	Coping: distraction	Parents distract themselves to cope
	Coping: focus	Parents do not focus on the disease to cope, they resume as if life was normal
	Coping: information	Parents cope by having more information
	Coping: monitoring	Parents find monitoring their health is a way of coping
	Coping: no coping	Parents could not cope with information
	Coping: positivity	Parent remains positive
	Coping: prognosis	Parents think knowing prognosis will help to cope
	Coping: risk factor	Parents think not labeling with a disease is a way to cope
Support Systems for Families	Support: facebook groups	Parents find support from Facebook Support Groups
	Support: family	Parents say that their family offers emotional support
	Support: other medical parent	Parent finds support from another parent who had a child who went through a different disease
	Support: other PD parents	Parents find support from other PD parents
	Support: PCP	Parents feel supported by their pediatrician
	Support: religion	Parents feel supported through their religion
	Support: social work	Parent had social worker for support
	Support: work	Parent feels supported by bosses and coworkers
Other/Uncategorized	Change In Behavior	Parent's behaviors has changed since diagnosis
	Change In Lifestyle	Parent's lifestyle has changes from diagnosis

	COVID	Parent talks about COVID-19 and pandemic
	Crutch	Parent thinks child may use diagnosis as crutch
	Curse	in vivo, parent says diagnosis is like a curse
	Difficult Days	in vivo, Parents still have difficult days of coping
	Family Planning	Parent discusses their decisions having more children
	Idle Hands	in vivo, Parent had idle hand during wait for diagnosis to Google information
	Infantile	Parent talks about infantile form
	Medical Background	Parent has medical background which influences understanding and self monitoring
	Postpartum Effects	Parent expressed emotions were stronger due to postpartum effects
	Proactive	Parent is proactive with children’s health and monitoring
	Research	Child is in a research study
